# Conserved presence of G-quadruplex forming sequences in the Long Terminal Repeat Promoter of Lentiviruses

**DOI:** 10.1038/s41598-017-02291-1

**Published:** 2017-05-17

**Authors:** Rosalba Perrone, Enrico Lavezzo, Giorgio Palù, Sara N. Richter

**Affiliations:** Department of Molecular Medicine, University of Padua, via Gabelli 63, 35121 Padua, Italy

## Abstract

G-quadruplexes (G4s) are secondary structures of nucleic acids that epigenetically regulate cellular processes. In the human immunodeficiency lentivirus 1 (HIV-1), dynamic G4s are located in the unique viral LTR promoter. Folding of HIV-1 LTR G4s inhibits viral transcription; stabilization by G4 ligands intensifies this effect. Cellular proteins modulate viral transcription by inducing/unfolding LTR G4s. We here expanded our investigation on the presence of LTR G4s to all lentiviruses. G4s in the 5′-LTR U3 region were completely conserved in primate lentiviruses. A G4 was also present in a cattle-infecting lentivirus. All other non-primate lentiviruses displayed hints of less stable G4s. In primate lentiviruses, the possibility to fold into G4s was highly conserved among strains. LTR G4 sequences were very similar among phylogenetically related primate viruses, while they increasingly differed in viruses that diverged early from a common ancestor. A strong correlation between primate lentivirus LTR G4s and Sp1/NFκB binding sites was found. All LTR G4s folded: their complexity was assessed by polymerase stop assay. Our data support a role of the lentiviruses 5′-LTR G4 region as control centre of viral transcription, where folding/unfolding of G4s and multiple recruitment of factors based on both sequence and structure may take place.

## Introduction

Lentiviruses are a genus of viruses that infect a broad range of mammalians, causing severe diseases mainly characterized by immunological and neurological deficiencies. They belong to the *Retroviridae* family: as such, they are characterized by a ssRNA genome that, once retrotranscribed by the viral reverse trascriptase enzyme, integrates into the host cell chromosome in the provirus form. The provirus can then undergo a productive replicative cycle or remain in a dormant state known as “latency”. Among lentiviruses, the Human Immunodeficiency Virus 1 (HIV-1) was first characterized in 1983^1, 2^ when it was proposed as the causative agent of the acquired immunodeficiency syndrome (AIDS). HIV-2, SIV (Simian Immunodeficiency virus) and FIV (Feline Immunodeficiency virus), together with HIV-1, are lentiviruses responsible for severe and often fatal acquired immunodeficiency syndrome-like diseases. Other lentiviruses, i.e. Visna/Maedi virus, equine infectious anemia virus (EIAV) and caprine arthritis/encephalitis virus (CAEV) cause different pathologies, such as neurological disorders, anemia and wasting or arthritis and encephalitis. Lentiviruses that infect cattle, such as Bovine Immunodeficiency virus (BIV) and Jembrana Disease Virus (JDV), result in further pathophysiologic events that range from asymptomatic to systemic acute diseases^3^. Despite the wide range of clinical effects of lentiviral infections and the high divergence in nucleotide (nt) composition of their genome^4^, all lentiviruses are similar in structure, genome organization, and mode of replication. Importantly, effective progression of the viral cycle relies on the proper function of the long terminal repeat (LTR): even if the LTR region varies in terms of length and composition, it always originates from the multi-step reverse transcription process, which makes 3 main LTR regions, namely U3, R and U5. Once integrated, the 5′-LTR, and in particular its U3 region which is characterized by transcription factor binding sites, serves as unique viral promoter^5^. Each lentivirus has peculiar cis-acting regulatory sequences that are both essential for promoter activity and different from each other^6^. For example, the HIV-1 LTR is composed of 3 main sub-regions: the core, where GC-rich binding sites for Sp1 are located; the enhancer, just upstream of the core, containing binding sites for NF-κB; a modulatory region that comprises binding sites for several transcription factors, including C/EBP factors.

In HIV-1, formation of multiple G-quadruplex (G4) structures in the viral and proviral genome^7, 8^, and in particular in the LTR promoter^9, 10^, has been reported. G4s are nucleic acids secondary structures that may form in single-stranded G-rich sequences under physiological conditions^11–13^. Four Gs bind via Hoogsteen-type hydrogen bonds base-pairing to yield G-quartets, which stack to form the G4. The presence of K^+^ cations specifically supports G4 formation and stability^14–16^. In eukaryotes G4s have been shown to be involved in key regulatory roles, including transcriptional regulation of gene promoters and enhancers, translation, chromatin epigenetic regulation, DNA recombination^9, 13, 17–19^. Expansion of G4-forming motifs has been associated with relevant human neurological disorders^20–26^. Formation of G4s *in vivo* has been consolidated by the discovery of cellular proteins that specifically recognize G4s^27, 28^ and the development of G4-specific antibodies^29, 30^.

The presence of G4s has been recently reported in viruses^31^, such as SARS coronavirus^32^, human papilloma, Zika, Ebola and hepatitis C genomes^33–36^, Epstein–Barr virus^37, 38^ and herpes simplex virus 1^39, 40^.

In HIV-1, functionally significant G4s have been implicated in pathogenic mechanisms^7–10, 27^. Formation of G4s in the U3 region of the 5′-LTR, the unique viral promoter, resulted in down-modulation of viral transcription. Further inhibition of viral transcription was achieved using G4 ligands^41, 42^ or by cellular proteins^27^. One LTR G4, LTR-IV, acted as a modulator of the dynamic G4s within the LTR region^43^.

Taking into account the strong evidence of a G4-mediated regulatory mechanism in the HIV-1 promoter, we here aimed at investigating the presence of putative G4 folding sequences (PQSs) in the LTR region of all other lentiviruses, phylogenetically correlate them and analyse their actual G4 formation. We showed that all primate lentiviruses have sequences capable of folding into G4 in the U3 region of their LTR promoter; as in HIV-1, the presence of G4s correlates with that of transcription factors binding sites, in particular Sp1 and NFκB. Our data indicate the 5′-LTR G4 region as a crucial control centre for viral transcription that was maintained during evolution of lentiviruses.

## Results

### Putative G-quadruplex forming sequences are present in the 5′-LTR of both human and non-human primate lentiviruses

**Figure 1:**
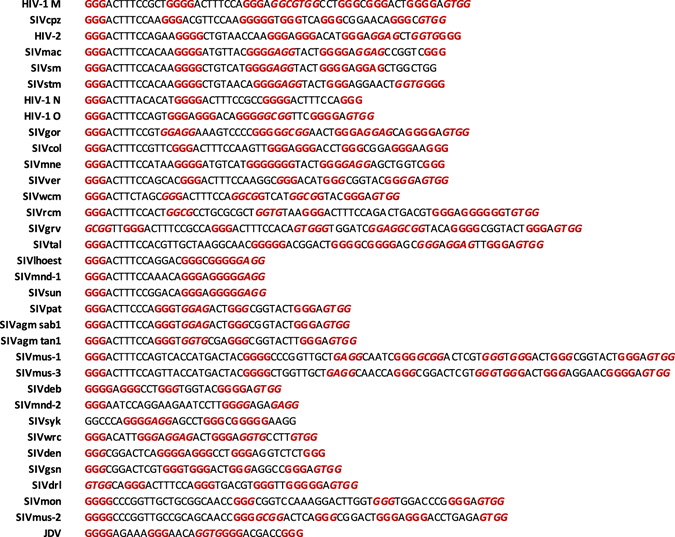
PQS analysis within the LTR region of lentiviruses (our reference HIV-1 group M is at the top of the list). GGG tracts are shown in red and bold; bulged tracts (e.g. GXGG that do not overlap with adjacent GGG tracts) are shown in red, bold and italics. Lower case letters that specify the SIV strain refer to the simian type they were first identified from (e.g. SIVcpz from chimpanzee or *Pan troglodytes troglodytes*, SIVsm from sooty mangabey monkey or *Cercocebus atys*). A complete list of PQS in all lentiviruses along with references and host species is provided in Supporting Table S1.

To check if the G4 forming region observed in the 5′-LTR of the HIV-1 provirus was a conserved feature of lentiviruses, we investigated the presence of putative G4 forming sequences (PQS) in the LTRs of all known lentiviruses. The viruses belonging to the lentivirus genus can be grouped according to their 5 host types. The primate group, which our reference HIV-1 group M belongs to, comprises viruses that naturally infect both human (HIV-1 and HIV-2) and non-human primates (Simian Immunodeficiency Virus, SIV) mainly belonging to the *Cercopithecidae* family^44^. The SIV sub-group is the most abundant and composed of viruses isolated from 41 different primate species. Among these, 29 SIV genomes that include the entire LTR region are available: these were considered in our analysis (Fig. 1 and Supporting Table S1). The ovine-caprine group includes 3 viruses: Visna/Maedi virus, Caprine Arthritis Encephalitis Virus (CAEV) and Ovine Lentivirus (OL); the bovine group consists of Bovine Immunodeficiency Virus (BIV) and Jembrana Disease Virus (JDV); the equine group is represented by Equine Infectious Anemia Virus (EIAV); the feline group comprises Feline Immunodeficiency Virus (FIV) that naturally infects members of the *Felidae* family (Supporting Table S1).

Analysis of PQS was initially performed using the online-based algorithm software (QGRS Mapper). The search was limited to 3-stacked tetrads G4s because these are the most abundant stable G4s within eukaryotic promoter regions^45^ and these were found in the HIV-1 group M LTR^9^. The following G4 pattern was thus investigated: GGG_≥3_N_0–12_GGG_≥3_N_0–12_GGG_≥3_N_0–12_GGG_≥3_, where N is a 0–12 nt-long loop sequence. This initial analysis allowed to identify the main G4 forming regions, which were next manually analysed in terms of G-tracts, loop composition and size. This step was necessary to include G4s with a single-nt bulge; in fact, this type of G4 has been previously found in the HIV-1 LTR-IV G4^43^. Two-stacked tetrads G4 structures were excluded from the analysis. Results are summarized in Fig. 1 and Supporting Table S1.

PQSs were found in the 5′-LTR regions of 97% viruses of the primate group (32 viruses over 33 analysed viruses). Interestingly, most G4 sequences were located in the U3 region of the viral LTRs, as previously found in the HIV-1 group M LTR^9^. Within the bovine group, JDV presented one LTR PQS formed by 4 tracts of 3 or more Gs, 1 GG tract and additional single Gs that could also be involved in G4 formation. Conversely, BIV and viruses of the equine, feline and ovine-caprine groups lacked the possibility to form three-stacked tetrad G4s in the LTR (Supporting Table S1). Some of these viruses presented sequences compatible with formation of two-stacked tetrad G4s or G4s involving multiple bulges (Supporting Table S2). These sequences were not further considered due to their low intrinsic stability and lack of preferred conserved location.

In the primate group, the identified LTR PQSs highly varied both in length (from 27 to 89 nts) and base composition, and were all characterized by several G-runs that could in principle form multiple overlapping G4s. The HIV-2, SIVcol, SIVcpz, SIVmac, SIVsm and SIVstm genomes, however, shared peculiar features with our reference HIV-1 group M LTR, such as length and the potential to form at least 3 stable G4 structures. In fact, the LTR PQSs of these viruses were all characterized by 4–6 tracts of 3 or more Gs, with additional GG tracts and interspersed Gs. Notably, SIVcpz LTR PQS was composed of 6 GGG tracts, 2 GG tracts and 1 G base that could form a single-nt bulge G4, exactly as we previously reported for the HIV-1 group M LTR^9, 43^. The only exception in the SIV group is SIVmnd2, the LTR PQS of which displayed multiple G-runs with the potential of atypical G4 folding, such as three-stacked tetrad G4s with 2-nt bulges. PQSs of HIV-1 groups N and O were also quite different, being shorter (43 nts) and with unique G-patterns (Fig. 1). Interestingly, in the primate group the full-length LTR sequence is only moderately conserved: the pairwise sequence similarity of all possible sequence pairs is reasonably low (mean = 56.70%, st.dev. = 6.25%, median = 55.59%) (Supporting Fig. 1a). In contrast, the possibility to form G4 is conserved even if the G4 pattern is different among strains (Supporting Fig. 1b).

### Putative G-quadruplex forming sequences of lentiviruses significantly overlap with Sp1 binding sites

We noted that most of the primate LTR G-rich sequences displayed a conserved sequence (GGGACTTTCC) located at the 5′-end of the PQS (Table 1) that corresponded to one NFκB binding site (consensus sequence 5′-GGGRNNYYCC-3′, R = A or G, N = any nt, and Y = C or T)^46^. The JDV PQS also partially overlapped with a NFκB binding site at its 3′ end (Table 1). In addition, the HIV-1 LTR G4s were associated to three Sp1 binding sites (consensus sequence KGGGCGGRRY, K = G or T, R = A or G and Y = C or T)^47^. Since Sp1 binding sites for only a very few viruses are available in the literature, the establishment of a straightforward correlation between LTR G4s and Sp1 was not possible. We thus used the online software PhysBinder^48^ to predict Sp1 binding regions in the LTR of all lentiviruses with relevant PQSs. In the primate lentiviruses, 30 out of 32 subgroups displayed Sp1 binding sites that overlapped with the PQS (Table 1). HIV-1 N and SIVwrc were two exceptions: the former was the only genome that displayed three NFκB binding sites overlapping with the PQS and no Sp1 binding site; the latter was the only genome that lacked both NFκB and Sp1 binding sites. All other subgroups presented 1–3 Sp1 binding sites associated to 0–2 NFκB binding sites, indicating a strong correlation between G4 and Sp1.

**Table 1:** Transcription factor binding sites in 5′-LTR PQS.

Group	Lentivirus	Transcription factor binding sites in LTR PQS	Ref
Primate	HIV-1 M	**GGGACTTTCC**GCT**GGGGACTTTCC**AGG*GAGGCGTGG*C*CTGGGCGGGA*C*TGGGGAGTGG*	49
	SIVcpz	**GGGACTTTCCAAGGGACGTTCC**AAGGGGGTGGGTCAGGGCGGAACAGGGCGTGG	
	HIV-2	**GGGACTTTCC**AGAAGGGGCTGTAACC*AAGGGAGGGAC*A*TGGGAGGAGC*T*GGTGGGG*	49
	SIVmac	**GGGACTTTCC**ACAA*GGGGATGTT*AC*GGGGAGGTA*CT*GGGGAGGAG*CC*GGTCGGG*	50
	SIVsm	**GGGACTTTCC**ACAAGGGGCTGTCATGGGGAGGTACTGGGGAGGAGCTGGCTGG	
	SIVstm	**GGGACTTTCC**ACAAGGGGCTGTAACAGGGGAGGTACTGGGAGGAACTGGTGGGG	
	HIV-1 N	**GGGACTTTAC**ACA**TGGGGACTTTCC**GCC**GGGGACTTTCC**AGGG	
	HIV-1 O	**GGGACTTTCC**AGTGGGAGGGACAGGGGGCGGTTCGGGGAGTGG	
	SIVgor	**GGGACTTTCC**GTGGAGGAAAGTCCCCGGGGGCGGAACTGGGAGGAGCAGGGGAGTGG	
	SIVcol	**GGGACTTTCC**GTT**CGGGACTTTCC**AAGTTGGGAGGGACCTGGGCGGAGGGAAGGG	
	SIVmne	**GGGACTTTCC**ATAAGGGGATGTCATGGGGGGGTACTGGGGAGGAGCTGGTCGGG	
	SIVver	**GGGACTTTCC**AGCA**CGGGACTTTCC** AAGGCGGGACATGGGCGGTACGGGGAGTGG	
	SIVwcm	**GGGACTTC**TAGC**GGGACTTTCC** AGGCGGTCATGGCGGTACGGGAGTGG	
	SIVrcm	**GGGACTTTCC**ACTGGCGCCTGCGCGCTGGTGTAA**GGGACTTTCC**AGACTGACGTGGGAGGGGGGTGTGG	
	SIVgrv	GCGGTT**GGGACTTT**CCGCCA**GGGACTTT**CCACAGTGGGTGGATCGGAGGCGGTACAGGGGCGGTACTGGGAGTGG	
	SIVtal	**GGGACTTTCC**ACGTTGCTAAGGCAACGGGGGACGGACTGGGGCGGGGAGCGGGAGGAGTTGGGAGTGG	
	SIVlhoest	**GGGACTTTCC**AGGACGGGCGGGGGAGG	
	SIVmnd-1	**GGGACTTTCC**AAACAGGGAGGGGGAGG	
	SIVsun	**GGGACTTTCC**GGACAGGGAGGGGGAGG	
	SIVpat	**GGGACTTCC**C*AGGGTGGAGACTGGGCGGTACTGGGAGTGG*	51
	SIVagm sab1	**GGGACTTTCC** AGGGTGGAGACTGGGCGGTACTGGGAGTGG	
	SIVagm tan1	**GGGACTTTCC** AGGGTGGTGCGAGGGCGGTACTTGGGAGTGG	
	SIVmus1	**GGGACTTTCC**AGTCACCATGACTACGGGGCCCGGTTGCTGAGGCAATCGGGGCGGACTCGTGGGTGGGACTGGGCGGTACTGGGAGTGG	
	SIVmus3	**GGGACTTTCC**AGTTACCATGACTACGGGGCTGGTTGCTGAGGCAACCAGGGCGGACTCGTGGGTGGGACTGGGAGGAACGGGGAGTGG	
	SIVdeb	GGGGAGGGCCTGGGTGGTACGGGGAGTGG	
	SIVsyk	GGCCCAGGGGAGGAGCCTGGGCGGGGGAAGG	
	SIVwrc	GGGACATTGGGAGGAGACTGGGAGGTGCCTTGTGG	
	SIVden	GGGCGGACTCAGGGGAGGGCCTGGGAGGTCTCTGGG	
	SIVgsn	GGGCGGACTCGTGGGTGGGACTGGGAGGCCGGGAGTGG	
	SIVdrl	GTGGCA**GGGACTTTCC**AGGGTGACGTGGGTTGGGGGAGTGG	
	SIVmon	GGGGCCCGGTTGCTGCGGCAACCGGGCGGTCCAAAGGACTTGGTGGGTGGACCCGGGGAGTGG	
	SIVmus2	GGGGCCCGGTTGCCGCAGCAACCGGGGCGGACTCAGGGCGGACTGGGAGGGACCTGAGAGTGG	
Bovine	JDV	GGGGAGAAAGGGAACAGGTGGGGACGACC**GGG(ACCTTTCC)**	

Binding sites for NFκB are in bold. Sp1 binding sites reported in the literature are indicated in italics and are underlined. Sp1 binding sites predicted by Physbinder are underlined.

### 5′-LTR PQSs are highly conserved among lentivirus isolates

**Figure 2:**
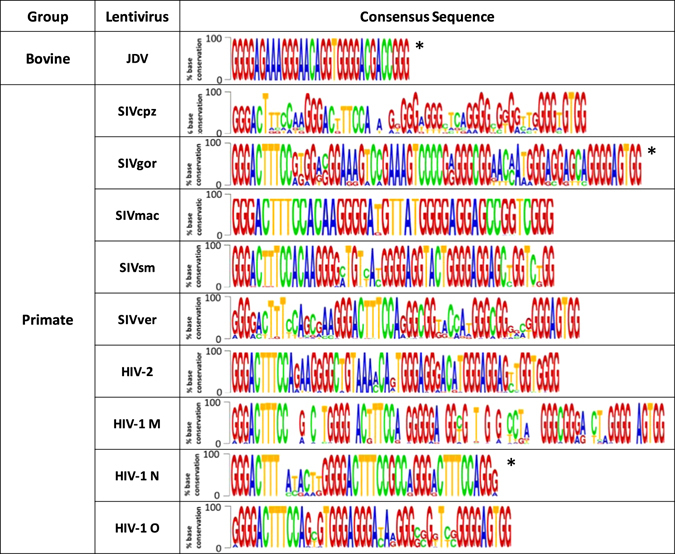
Base conservation of G4 sequences among isolates of lentiviruses. *Consensus sequence obtained by the alignment of 6–10 sequences. All other consensus sequences were obtained by alignment of more than 10 sequences. Lentiviruses with identified PQSs that are not present in this table had lower than 5 complete reported LTR sequences.

The relevance of the identified PQSs within the viral context was established by assessing the degree of base conservation among different isolates of the same virus strain (Supporting Table S3). Only viruses with more than 5 complete LTR sequences and from different strains were considered. An extremely high degree of G-base conservation, especially within G-tracts, was found for almost all 5′-LTR PQSs (generally higher than 70% and in most cases higher than 90%) (Fig. 2). In particular, the potentiality to form G4 was maintained in all viruses.

### The 5′-LTR PQSs are present in phylogenetically related lentiviruses

Because the identified PQGs were quite diverse among viruses infecting different hosts, with the few exceptions highlighted above, we checked the level of sequence identity/variation among lentiviruses that occurred during evolution. To this end, we built a phylogenetic tree based on the alignment of the *pol* gene sequence of lentiviruses. The pol sequence-based tree was chosen because it displayed higher bootstrap support than the LTR-based tree: it showed some minor differences in the branching order with the LTR-based tree, but it correctly grouped the non-primate lentiviruses outside the primate group^52^ and resulted consistent with previously reported phylogenetic analysis^53^. This phylogenetic analysis confirmed that the host-based groups (Fig. 1 and Supporting Table S1) were correctly assigned since phylogenetically divergent.

**Figure 3:**
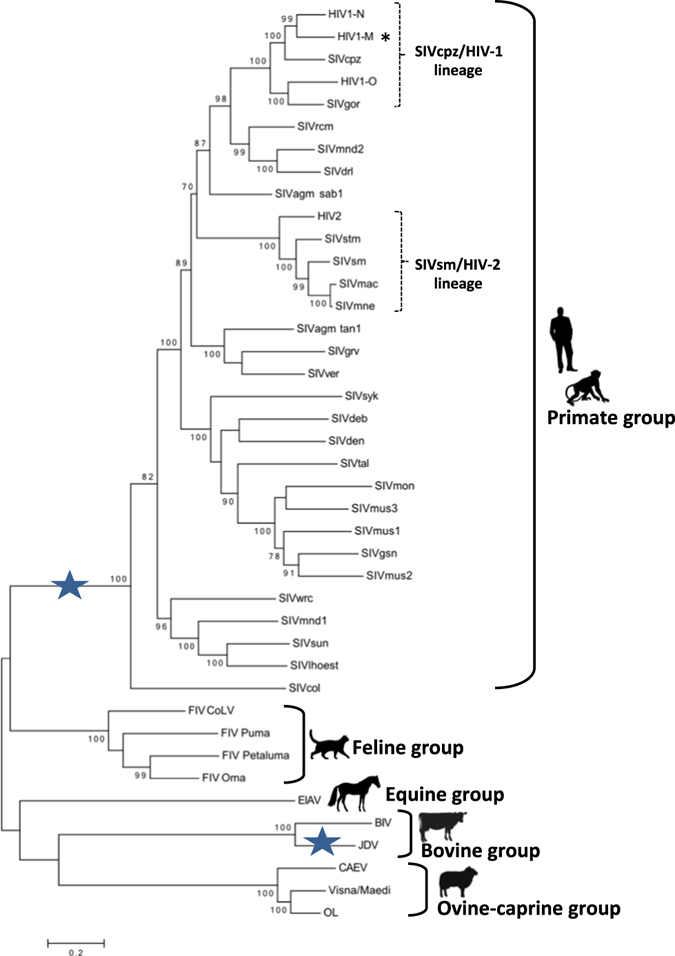
Phylogenetic tree of lentiviruses based on the *pol* gene. Blue stars correspond to the presence of PQSs in the corresponding group of lentiviruses. The symbol * indicates our reference virus HIV-1 group M. Human and animal silhouettes were obtained at http://www.freepik.com and http://www.flaticon.com/ (http://www.freepik.com/free-vector/business-team-outlines-pack_831669.htm#term=human; http://www.flaticon.com/free-icon/monkey_47138; http://www.freepik.com/free-vector/cat-silhouettes-set_718091.htm#term=cat; http://www.flaticon.com/free-icon/horse-standing-black-shape_35907; http://www.freepik.com/free-vector/cows-and-bull-silhouettes_788343.htm; http://www.freepik.com/free-vector/pack-of-farm-animal-silhouettes_1058750.htm#term=sheep&page=1&position=29).

The primate group, comprising our reference virus HIV-1 group M (symbol * in Fig. 3), included viruses that naturally infect both human (HIV-1 and HIV-2) and non-human primates (SIVs). An interesting feature here was the crossover of HIV and SIV lineages that indicates multiple cross-species transmission from simian to humans: in particular, SIVcpz from chimpanzee or *Pan troglodytes troglodytes* was the most closely related to HIV-1 group M, whereas SIVsm from sooty mangabey monkey or *Cercocebus atys* was closer to HIV-2^54^. HIV-1 groups M and N originated from independent jumps from chimpanzee^55^ and HIV-1 group O from the western gorilla subspecies *Gorilla gorilla* ^56^, resulting in HIV-1 strains with very different pathogenic potential: HIV-1 group M causes the well-known AIDS pandemic, groups N and O cause a milder disease and have been limited to a few individuals mostly in Cameroon^57, 58^. This phylogenetic analysis on one hand further supports the central role of G4 formation in the LTR, since this feature was maintained in multiple and unrelated cross-species transmission from a simian ancestor strain, and it explains why all primate lentiviruses display PQSs in the same LTR U3 region; on the other it gives reason of the substantial differences in PQS among strains.

Interestingly, only JDV of the bovine group displayed LTR PQSs, even if it was phylogenetically more distant to primate lentiviruses than the feline group, which lacked three-stacked tetrad LTR PQSs (blue stars in Fig. 3).

### The identified 5′-LTR PQSs can fold into G4 structures

The actual ability of PQSs to form G4 structures was next investigated by *Taq* polymerase stop assay. Representative PQSs from the primate group and the unique JDV PQSs were analysed to cover the widest phylogenetic distance: PQSs from the SIVcpz/HIV-1 lineage (our reference HIV-1 group M, SIVcpz, HIV group N, HIV group O and SIVgor), the SIVsm/HIV-2 lineage (HIV-2, SIVmac and SIVsm) and SIVlhoest, SIVwrc, SIVsyk and SIVagm sab1, which are progressively closer to HIV-1 M in the phylogenetic tree, were selected.

**Figure 4:**
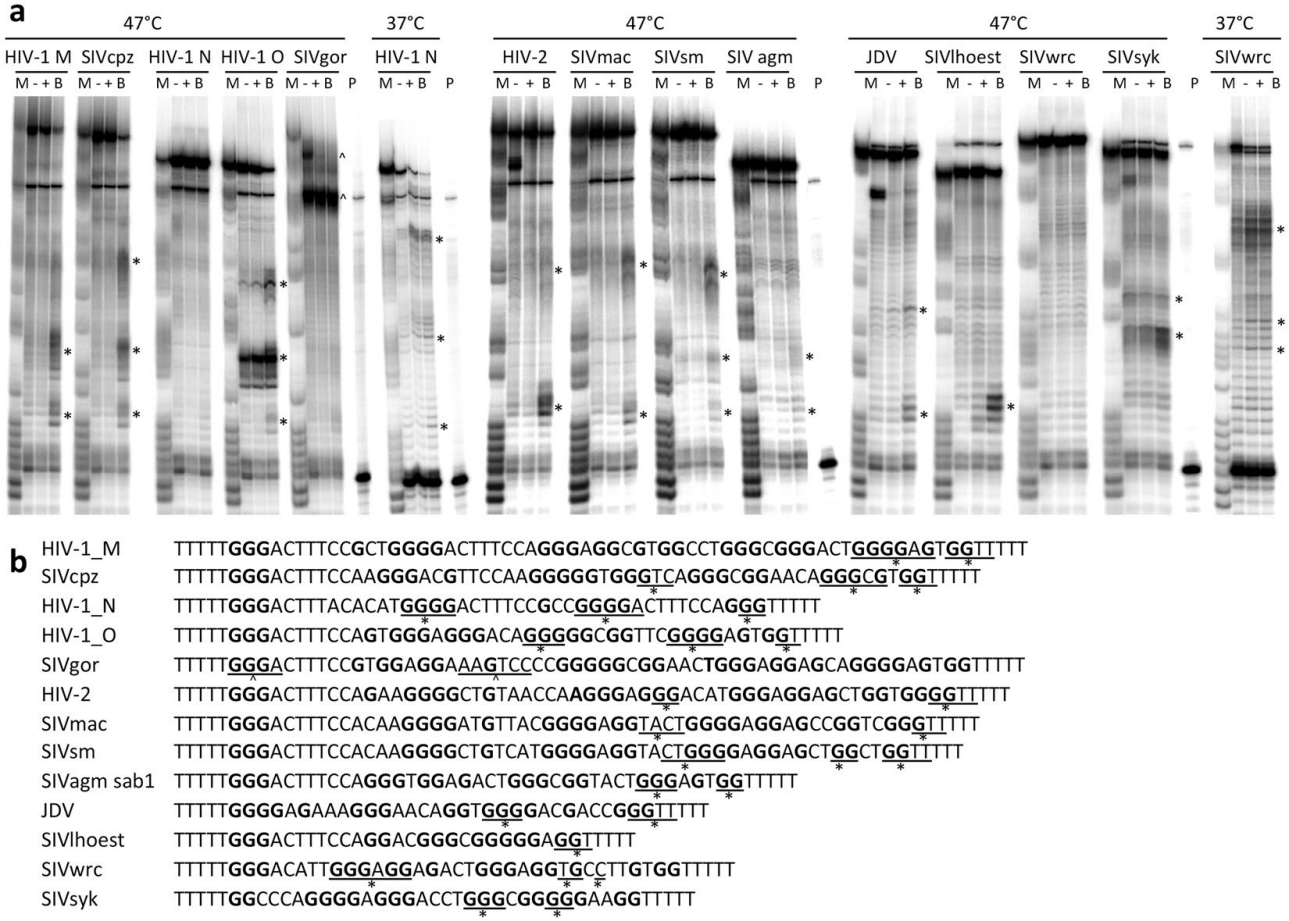
*Taq* polymerase stop assay of lentiviral G4 sequences. Oligonucleotides bearing PQSs were folded in the absence (−) or presence (+) of KCl. KCl-treated samples were further incubated with the G4 ligand BRACO-19 (B). Oligonucleotides were used as templates in a *Taq* polymerase reaction at 47 °C or 37 °C as indicated. Symbols * indicate premature stop site at G bases in gel images (**a**) and in the corresponding G4 sequences (**b**). Symbols ^ indicate stop sites independent of G4 folding. G-bases are shown in bold.

The chosen sequences were investigated in the absence/presence of K^+^ to establish G4 formation, and in the presence of the G4 ligand BRACO-19 (200 nM) to assess ligand-induced G4 formation, which has been reported in the case of HIV-1 group M^9^. In the presence of K^+^ and BRACO-19, premature stop sites appeared in most of the selected PQSs (Fig. 4a) and corresponded to the most 3′-G-tract involved in G4 formation (Fig. 4b). In all cases, G4-specific stops were more pronounced in the presence of the G4 ligand (compare lanes + with lanes B in Fig. 4a) indicating that these G4s can be stabilized and in some cases induced by specific G4 ligands, consistently with previously reported data^9^. Sequences where no G4-related stops were obtained during the *Taq* polymerase elongation step at 47 °C, i.e. HIV N and SIVwrc (Fig. 4a), were further analysed at elongation at 37 °C to solve thermodynamically less stable secondary structures. In these conditions, these two sequences folded in G4s as well, as indicated by specific stops at the main G-tracts (Fig. 4a,b). Only SIVlhoest PQS folded in a unique G4 species, while all other tested sequences folded in 2 or 3 different G4s (Fig. 4a,b). Interestingly, PQSs of HIV-1 group M and SIVcpz not only were similar in terms of number of G-runs (Fig. 1, Table 1 and Supporting Table S1), conserved (Fig. 2) and phylogenetically related (Fig. 3), but were both also able to fold in 3 very similar and mutually exclusive G4 structures (Fig. 4a). Indeed, the predicted G4 species of SIVcpz fully coincided with those of HIV-1 group M^9, 43^ (Fig. 4b). PQSs in the 5′-LTR of HIV-1 group O and group N were also able to fold in G4 even if HIV-1 group N G4s were probably less stable because they could be visualized only at the lower *Taq* polymerase elongation temperature. Unfortunately, it was not possible to investigate SIVgor G4s by *Taq* Polymerase Stop assay because an extremely stable premature stop that did not correspond to a G4 structure was present (Fig. 4a,b). This stop was probably due to a hairpin-like secondary structure with 10 base pairs, as predicted by a web-based DNA tool (https://www.idtdna.com/calc/analyzer). As for viruses in the SIVsm/HIV-2 lineage, the behaviour of HIV-2, SIVmac and SIVsm G4s were extremely similar and in accordance to their relatedness in terms of sequence (Fig. 1) and evolutionary history (Fig. 3): they could effectively fold in at least two different G4s, the major one of which was located at the 3′-end of the sequence (Fig. 4b) and mainly induced by the addition of BRACO-19 (Fig. 4a).

## Discussion

The G4 cluster has been previously shown to be a fine modulator of HIV-1 group M transcription: in particular, when HIV-1 LTR G4s fold, transcription is inhibited^9^. Formation of HIV-1 LTR G4s is modulated by interaction with different cellular proteins, i.e. nucleolin and hnRNPA2/B1, which further inhibit or release transcription by inducing or unfolding G4s, respectively^27, 59^. The existence of cellular proteins that specifically interact with the HIV-1 G4 system is a powerful indication of the actual formation of LTR G4s *in vivo*.

A further indication comes from the present work, showing that almost all primate lentiviruses and JDV, a cattle infective virus, display G4 forming regions in the U3 region of their 5′-LTR promoter. These regions and their ability to fold into G4 are extremely well conserved. The LTR G4s are also evolutionary related: on one hand the most similar LTR G4 regions (i.e. HIV-1 group M and SIVcpz, HIV-2 and SIVmac/SIVsm) belong to viruses that are phylogenetically the closest; on the other, LTR G4s in primate lentiviruses, while sharing the possibility to form G4, are diverse in sequence, possibly because of the early divergence of the different lineages from a common ancestor (Fig. 3).

A further note of interest is that, while the feline lentiviruses that are phylogenetically closer to the primate lentiviruses do not possess naturally folding G4 regions, the more distantly related JDV of the bovine group do. In general, however, these latter G-rich regions form low complexity G4s, in contrast to primate G4s that are multiple, overlapping and thus mutually exclusive^9^. We have suggested that the HIV-1 group M LTR G4 complexity is necessary for the tuning of G4 modulation: in particular, initial evidence indicated that the least stable HIV-1 G4, LTR-IV, may be required to release the inhibitory activity of the most stable HIV-1 G4, LTR-III^43^. It is thus possible that the primate LTR G4s are more evolutionary progressed and control viral transcription in a G4-based high-complexity mechanism.

Our phylogenetic analysis showed that LTR G4s have evolved independently of the common ancestor in the primate and bovine group. This fact indicates that the presence of a G4-based transcription control must be beneficial to the overall virus biology so that it has been selected during lentivirus evolution. In addition, 6 out of 9 viruses that lacked the presence of three-stacked tetrad G4s, displayed sufficiently clustered G tracts to allow the formation of less stable two-stacked tetrad G4s (Supporting Table S2). This might be indication of the initial evolution towards more stable G4s also in these viruses.

Based on the evidence that cellular proteins are required for the LTR G4 modulatory mechanism^27^, a further possible explanation for LTR G4 diversity in lentiviruses is that the selection of G4 in the LTR promoter has been driven by the presence/absence of the necessary host co-factors. This would explain the LTR G4s host-specificity observed in the present work.

In this direction, we have found a significant correlation between the presence of G4s and Sp1 binding sites in the LTR promoter of lentiviruses. Sp1 is a ubiquitous transcription factor that has been shown to be the main driver of HIV-1 basal transcription through binding to the three sites in the U3 region of the 5′-LTR^60^. Beside the duplex, Sp1 is able to bind DNA in its G4 conformation, both in eukaryotic cells^61^ and HIV-1^8^. Our data are in line with the previously reported association between G4s and Sp1 binding sites in human genes^62^. The reported suppression of viral gene expression when Sp1 binding to the HIV-1 5′-LTR is disrupted by cellular proteins or gene editing^63, 64^ may further support a key role played by G4s as regulatory elements of viral transcription.

One or more NFκB sites were also generally present upstream of the G4-forming region. In HIV-1 group M, we have previously shown that the sequence comprising NFκB, which could in principle fold into an additional overlapping G4, does not fold *in vitro* in the presence of K^+^ or G4 ligands^9^. Considering the degree of conservation of this sequence just upstream of the Sp1 binding sites and the G4 folding region, we suggest two possibilities: i) the recruitment of NFκB is necessary for processes that occur at the downstream G4 region; ii) there are additional cellular factors that induce G4 folding at this region. The former hypothesis is supported by the reported interaction of NFκB and Sp1 in an orientation and position-dependent manner^65^, which, based on our present observations, may rely on G4 folding/unfolding equilibria. The latter hypothesis is supported by the observation that nucleolin, the major reported LTR G4 binding protein^27^, preferentially binds regions that form low stability G4s^66^. The effect of G4-inducing proteins is expected to be more pronounced in less intrinsically stable G4s regions, such as the NFκB binding site, and thus biologically more significant.

On the whole, even if lentiviruses are characterized by a rapid evolution rate, they present a G-rich region in the 5′-LTR that is evolutionary very conserved in terms of structure, but not of sequence. This feature is shared with other key viral elements, such as the Lys-tRNA primer-binding site (PBS) that is required to start reverse transcription^67^. Thus, the use of structural conserved elements in a mechanosensor-regulated mechanism appears a theme commonly exploited by lentiviruses to control crucial viral steps. A similar G4/iMotif mechanism has been recently proposed in the promoter of the c-myc oncogene^68^.

**Figure 5:**
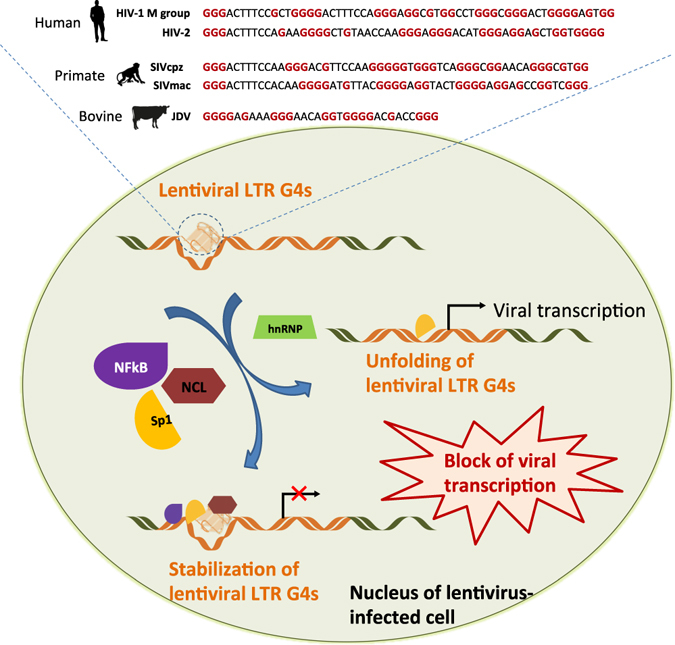
Model of transcription regulation in lentiviruses based on the 5′-LTR G4s. Sp1 and NFkB are transcription factors, NCL stands for the cellular protein nucleolin^27^, hnRNP stands for heterogeneous nuclear ribonucleoproteins. Human and animal silhouettes were obtained at http://www.freepik.com and http://www.flaticon.com/ (http://www.freepik.com/free-vector/business-team-outlines-pack_831669.htm#term = human; http://www.flaticon.com/free-icon/monkey_47138; http://www.freepik.com/free-vector/cows-and-bull-silhouettes_788343.htm).

In conclusion, we propose the 5′-LTR G4 region of lentiviruses as a control centre of viral transcription, where alternate folding/unfolding of the G4s and multiple recruitment of factors based on both sequence and structure may take place (Fig. 5).

## Materials and Methods

### G4 analysis of the lentivirus LTR Region

The LTR region of lentiviruses was analysed by QGRS Mapper (http://bioinformatics.ramapo.edu/QGRS/index.php) for prediction of G4 forming sequences. The following restrictions were applied: maximum length 45 nt; minimum G-group size 3 nt; loop size 0–12 nt.

### Analysis of sequence conservation of lentiviral LTRs and G4 patterns within the primate group

Complete LTR sequences, when available, were extracted from lentiviral strains belonging to the primate group. A multiple alignment was built using USEARCH^69^, followed by a manual editing to correct artefacts due to the low similarity among sequences (Supplementary Figure S1). The global sequence similarity of the alignment was calculated by averaging the percentage of similarity of all possible pairwise comparisons.

### Base conservation analysis of predicted G4 forming sequences

Predicted G4 forming sequences were further analysed in terms of base conservation by aligning sequences from Pubmed or from the HIV database (http://www.hiv.lanl.gov/) using USEARCH^69^. Accession numbers of the whole set of sequences were reported in Supplementary Table S3. The conservation analysis was performed only on lentiviruses with more than 5 sequences available in databases. LOGO representation of base conservation was obtained by the WebLogo software^70^.

### Prediction of transcription factor binding sites

The prediction of Sp1 binding sites in putative G4 forming sequences were performed by the web-based tool PhysBinder using the model HSA0000031.1 [SP1] with the Max. F-measure threshold (2 × True Positives/(2 × True Positives + False Positives + False Negatives)^48^.

### Molecular phylogenetic analysis of lentiviruses

The evolutionary history was inferred by using the Maximum Likelihood method based on the General Time Reversible mode^71^. The analysis involved 41 nucleotide sequences of the *pol* gene extracted from different lentiviruses (relative accession numbers in Table 1), which were multiple aligned with clustalW^72^. The percentage of trees in which the associated taxa clustered together is shown next to the branches (for values > = 70 on 500 bootstrap replicates^73^) (Fig. 3). The tree is drawn to scale, with branch lengths measured in the number of substitutions per site. All positions containing gaps and missing data were eliminated. There were a total of 2380 positions in the final dataset. Evolutionary analyses were conducted in MEGA6^74^.

### *Taq* Polymerase Stop Assay

*Taq* polymerase stop assay was performed as previously described^9^. Briefly, the 5′-end labeled primer was annealed to its template (Supporting Information, Table S1) in lithium cacodylate buffer in the presence or absence of KCl 100 mM and by heating at 95 °C for 5 min and gradually cooling to room temperature. Where specified, samples were incubated with BRACO-19 (200 nM). Primer extension was conducted with 2 U of AmpliTaq Gold DNA polymerase (Applied Biosystem, Carlsbad, California, USA) at 47 °C or 37 °C for 30 min. Reactions were stopped by ethanol precipitation, primer extension products were separated on a 15% denaturing gel, and finally visualized by phosphorimaging (Typhoon FLA 9000).

## Electronic supplementary material

Supplementary info
