# Supplementary material for: Conserved presence of G-quadruplex forming sequences in the Long Terminal Repeat Promoter of Lentiviruses
Source: Sci Rep. 2017 May 17;7:2018. doi: 10.1038/s41598-017-02291-1 (PMC5435695; doi:10.1038/s41598-017-02291-1)
Supplement: Supplementary file 1 — Supplementary info [file 41598_2017_2291_MOESM1_ESM.pdf]

Conserved presence of G-quadruplex forming sequences in the Long Terminal Repeat Promoter of Lentiviruses

Rosalba Perrone, Enrico Lavezzo, Giorgio Palù, Sara N. Richter*

Department of Molecular Medicine, University of Padua, via Gabelli 63, 35121 Padua, Italy
*Corresponding author

Supplementary Figure S1. Multiple sequence alignment of LTRs from lentiviruses belonging to the primate group. a) Overview of the whole LTR region produced with Jalview [Waterhouse AM, Procter JB, Martin DMA, Clamp M, Barton GJ (2009) Jalview Version 2-a multiple sequence alignment editor and analysis workbench. Bioinformatics 25: 1189-1191. doi:10.1093/bioinformatics/btp033]. Predicted G4 motifs are highlighted in black, while the following colour code has been applied to nucleotides: adenine= green, thymine=blue, cytosine=orange, guanine=red. b) Inset of the G4 region in the multiple alignment, where the conservation of small blocks (but not of the whole G4 patterns) is evident.

a)

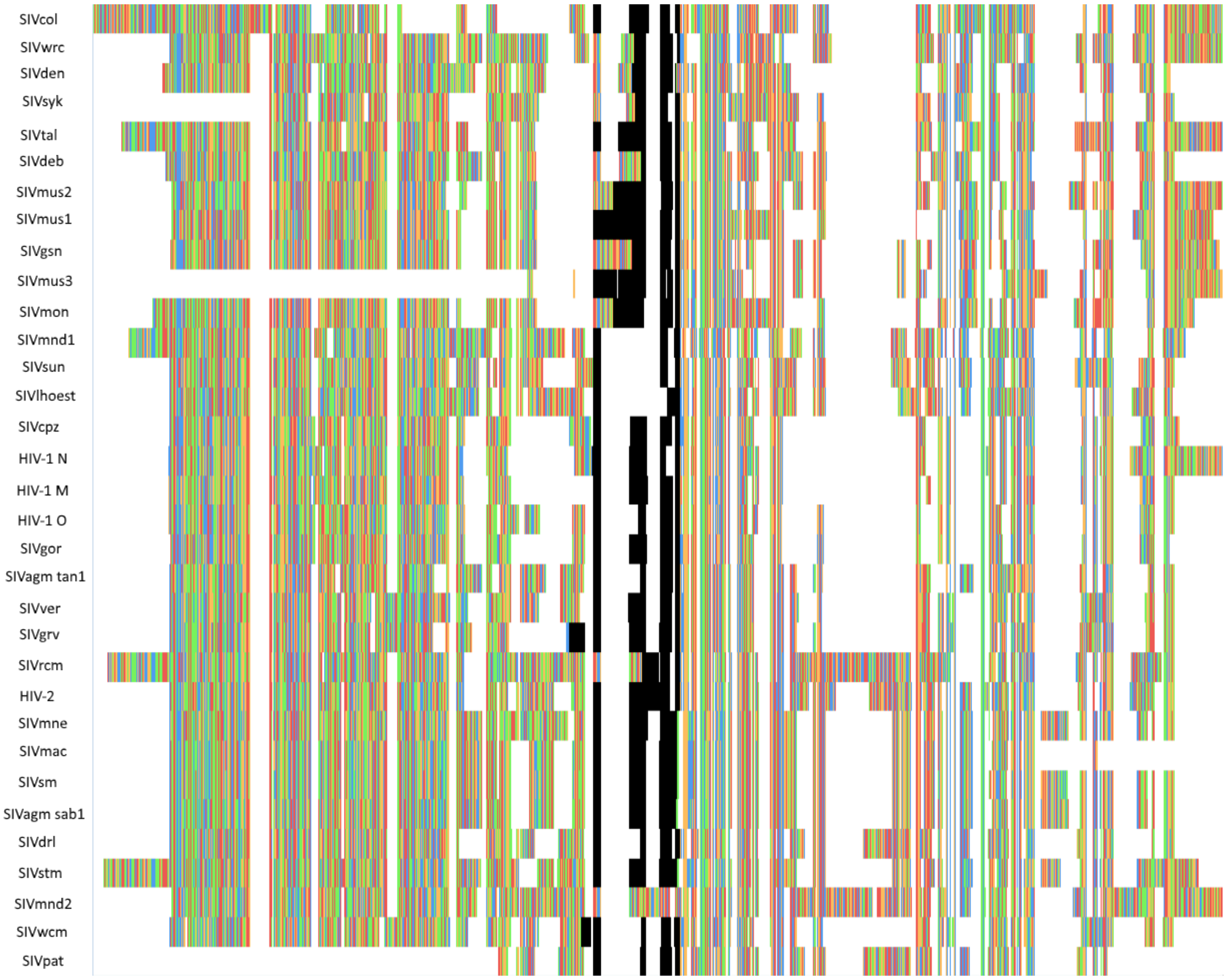

b)

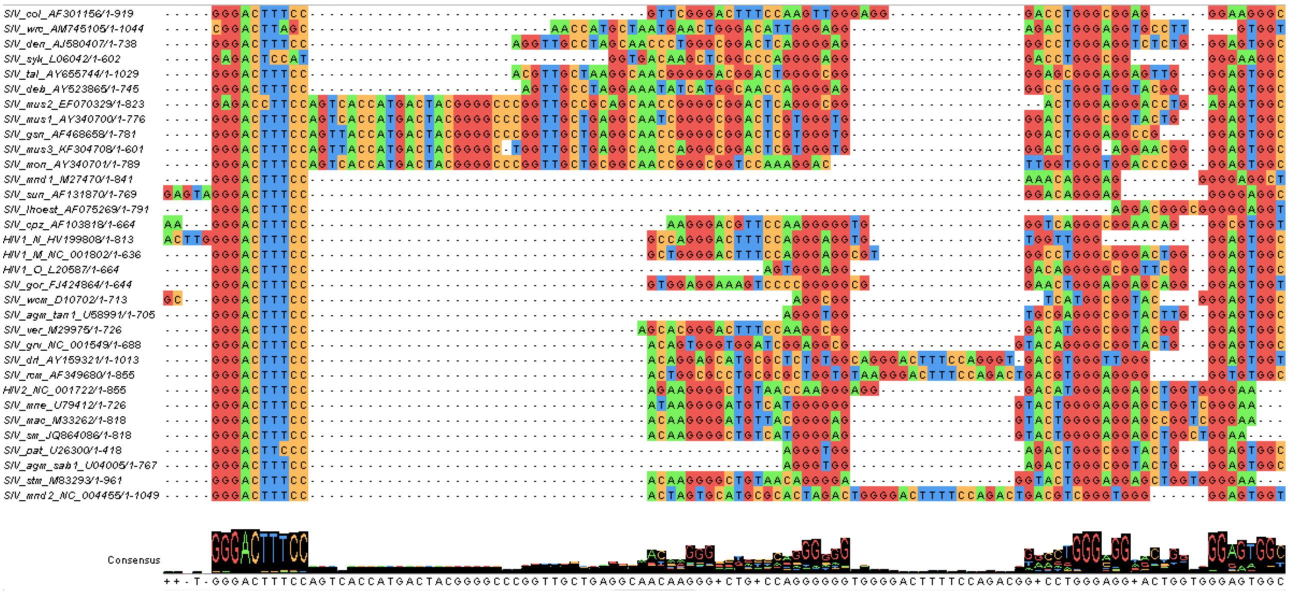

Supp. Table S1. PQS analysis within the LTR region of lentiviruses (our reference HIV-1 group M is highlighted in bold). GGG tracts are shown in red and bold; bulged tracts (e.g. GXGG that do not overlap with GGG tracts) are shown in red, bold and italics. The symbol * indicates host species for the primate group only.

Group	Lentivirus	Host species*	Accession number	PQS in the 5'-LTR	Length (nt)
Equine	EIAV		NC_001450.1	-	
Feline	FIV Petaluma		NC_001482.1	-	-
	FIV CoLV		EF455615.1	-	-
	FIV Puma		U03982	-	-
	FIV Oma		U56928	-	
Ovine-caprine	Visna/Maedi virus		NC_001452.1	-	-
	CAEV		NC_001463.1	-	-
	OL		NC_001511.1	-	-
Bovine	BIV		NC_001413.1	-	-
	JDV		U21603.1	GGGGAGAAAGGGAACAGGTGGGGACGACCGGG	32
Primate	**HIV-1 M**	**Homo sapiens**	**NC_001802.1**	**GGGACTTTCCGCTGGGGACTTTCCAGGGAGGCGTGGCCTGGGCGGGACTGGGGAGTGG**	***58***
	HIV-1 N	Homo sapiens	DQ017383	GGGACTTTACACATGGGGACTTTCCGCCGGGGACTTTCCAGGG	43
	HIV-1 O	Homo sapiens	L20587.1	GGGACTTTCCAGTGGGAGGGACAGGGGGCGGTTCGGGGAGTGG	43
	HIV-2	Homo sapiens	NC_001722.1	GGGACTTTCCAGAAGGGGCTGTAACCAAGGGAGGGACATGGGAGGAGCTGGTGGGG	56
	SIV col	Colobus Monkey	AF301156	GGGACTTTCCGTTCGGGACTTTCCAAGTTGGGAGGGACCTGGGCGGAGGGAAGGG	55
	SIV cpz	Chimpanzee	AF103818.1	GGGACTTTCCAAGGGACGTTCCAAGGGGGTGGGTCAGGGCGGAACAGGGCGTGG	54
	SIV deb	DeBrazza's Monkey	AY523865	GGGGAGGGCCTGGGTGGTACGGGGAGTGG	29

	SIV den	Dent's Monkey	AJ580407	GGGCGGACTCAGGGGAGGGCCTGG GAGGTCTCTGGG	36
	SIV drl	Drill Monkey	AY159321	GTGGCAGGGACTTTCCAGGGTGAC GTGGGTTGGGGGAGTGG	41
	SIV gor	Gorilla	FJ424864	GGGACTTTCCGTGGAGGAAAGTCCC CGGGGGCGGAACTGGGAGGAGCAG GGGAGTGG	57
	SIV grv	Grivet	NC_001549	GCGGTTGGGACTTTCCGCCAGGGAC TTTCCACAGTGGGTGGATCGGAGGC GGTACAGGGGCGGTACTGGGAGTG G	75
	SIV gsn	Greater Spot-nosed Monkey	AF468658	GGGCGGACTCGTGGGTGGGACTGG GAGGCCGGGAGTGG	38
	SIV lhoest	L'Hoest's Monkey	AF075269.1	GGGACTTTCCAGGACGGGCGGGGG AGG	27
	SIV mac	Macaque	M33262.1	GGGACTTTCCACAAGGGGATGTTA CGGGGAGGTACTGGGGAGGAGCCG GTCGGG	54
	SIV mnd-2	Mandrill	NC_004455.1	GGGAATCCAGGAAGAATCCTTGGG GAGAGAGG	32
	SIV mnd-1		M27470	GGGACTTTCCAAACAGGGAGGGGG AGG	27
	SIV mne	Pig-tailed Macaque	U79412	GGGACTTTCCATAAGGGGATGTCA TGGGGGGGTACTGGGGAGGAGCTG GTCGGG	54
	SIV mon	Mona Monkey	AY340701	GGGGCCCGGTTGCTGCGGCAACCGG GCGGTCCAAAGGACTTGGTGGGTGG ACCCGGGGAGTGG	64
	SIV mus-1	Mustached Monkey	AY340700	GGGACTTTCCAGTCACCATGACTAC GGGGCCCGGTTGCTGAGGCAATCGG GGCGGACTCGTGGGTGGGACTGGG CGGTACTGGGAGTGG	89
	SIV mus-2		EF070329	GGGGCCCGGTTGCCGCAGCAACCGG GGCGGACTCAGGGCGGACTGGGAG GGACCTGAGAGTGG	63
	SIV mus-3		KF304708	GGGACTTTCCAGTTACCATGACTAC GGGGCTGGTTGCTGAGGCAACCAG GGCGGACTCGTGGGTGGGACTGGG AGGAACGGGGAGTGG	88
	SIV pat	Patas	U26300	GGGACTTCCCAGGGTGGAGACTGG	41

		Monkey		GCGGTACTGGGAGTGG	
	SIV rcm	Red-capped Mangabey	AF349680	GGGACTTTCCACTGGCGCCTGCGCGCTGGTGTAAGGGACTTTCCAGACTGACGTGGGAGGGGGGTGTGG	70
	SIV agm sab1	Sabaeus Monkey	U04005.1	GGGACTTTCCAGGGTGGAGACTGGGCGGTACTGGGAGTGG	40
	SIV sm	Sooty Mangabey	JQ864086.1	GGGACTTTCCACAAGGGGCTGTCATGGGGAGGTACTGGGGAGGAGCTGGCTGG	53
	SIV stm	Stump-tailed Macaque	M83293	GGGACTTTCCACAAGGGGCTGTAACAGGGGAGGTACTGGGAGGAACTGGTGGGG	54
	SIV sun	Sun-tailed Monkey	AF131870.1	GGGACTTTCCGGACAGGGAGGGGGAGG	27
	SIV syk	Sykes' Monkey	L06042.1	GGCCCAGGGGAGGAGCCTGGGCGGGGGAAGG	31
	SIV tal	Talapoin Monkey	AY655744	GGGACTTTCCACGTTGCTAAGGCAACGGGGGACGGACTGGGGCGGGGAGCGGGAGGAGTTGGGAGTGG	68
	SIV agm tan1	Tantalus Monkey	U58991.1	GGGACTTTCCAGGGTGGTGCGAGGGCGGTACTTGGGAGTGG	41
	SIV ver	Vervet	M29975	GGGACTTTCCAGCACGGGACTTTCCAAGGCGGGACATGGGCGGTACGGGGAGTGG	55
	SIV wcm	White-crowned Mangabey	D10702	GGGACTTCTAGCGGGACTTTCCAGGCGGTCATGGCGGTACGGGAGTGG	49
	SIV wrc	Western Red Colobus	AM745105	GGGACATTGGGAGGAGACTGGGAGGTGCCTTGTGG	35

Supp Table S2. PQS analysis within the LTR region of lentiviruses that do not present three-stacked tetrad G4s (Table 1). GG, GGG and GXGG tracts are shown in bold; tracts that might be involved in G4 formation, according to the length of the linker/loop regions and the number of sufficiently close G-tracts, are additionally shown in red.

Group	Lentivirus	Accession number	5'-LTR (U3-R-U5)
Equine	EIAV	NC_001450.1	T**GTGGGG**TTTTTAT**GAGGGG**TTTTATAAATGATTATAAGAGTAAA<br>AAGAAAGTTGCTGATGCTCTCATAACCTTGTATAACCCAAA**GG**ACT<br>AGCTCATGTTGCTA**GG**CAACTAAACCGCAATAACCGCATTTGTGAC<br>GCGAGTTCCCCATT**GGTG**ACGCGTTAACTTCCTGTTTTTACAGTAT<br>ATAAGTGCTTGTATTCTGACAATT**GGG**ACTCAGATTCT**GCGG**TCTG<br>AGTCCCTTCTCTGCT**GGG**CTGAAAA**GG**CCTTTGTAATAAATATAAT<br>TCTCTACTCAGTCCCTGTCTCTAGTTTGTCTGTTCGAGATCCTACA
Feline	FIV Petaluma	NC_001482.1	T**GGG**ATGAGTATT**GG**AACCCTGAAGAAATAGAAAGAATGCTTAT**G**<br>**G**ACTA**GGG**ACTGTTTACGAACAAATGATAAAA**GG**AAATAGCTGAG<br>CATGACTCATAGTTAAAGCGCTAGCAGCTGCCTAACCGCAAAACCA<br>CATCCTAT**GG**AAAGCTTGCTAATGACGTATAAGTTGTTCCATTGTA<br>AGAGTATATAACCAGTGCTTTGTGAAACTTCGA**GG**AGTCTCTTTGT<br>T**GAGG**ACTTTTGAGTTCTCCCTT**GAGG**CTCCCACAGATACAATAAA<br>TATTTGAGATTGAACCCTGTCGAGTATCTGTGTAATCTTTTTTACC<br>TGT**GAGG**TCTC**GG**AATCC**GGG**CCGAGAACTTCGCA
	FIV CoLV	EF455615.1	T**GGG**A**GG**AAT**GG**AATTCAGTCTTAGATATAAAGAATAGAATGAAA<br>CTTGTATTAGAATA**GGTG**AGTCAGAGCTAGCTTAACCACAAACCGC<br>AAGTGCGAAC**GG**TTGAATAACCACATTTATGCTTGCATGCAAGAAA<br>TGACGTA**GGG**AATATCCTGTTGAGCCCTTTAAAAACCCTTGCAGTA<br>GACTGTAAGAGC**GG**CTAGTCT**GG**AA**GG**CTTC**GG**CTGACA**GG**CTGCC<br>CCTCA**GG**TAGAATAAAGCCCTTGAGAATTGAACCCTGACATCTGCC<br>TGAGAGTATTTTCTTAT**GTGGG**TCTAA**GGG**ATCC**GGG**TCTTA**GG**C<br>TCGCAG
	FIV Puma	U03982	T**GGG**AAGAGATTAATTTAGA**GG**CTGAAAGTTTAGAAGAACTAGAT<br>CTCATATAATTGTT**GG**CTGTAAGAAAGAAATATAGCTTTAAGAGTG<br>ACGCAAGTAACTAGCTTAACCGCAAACCGCAGATAAAACCCACATC<br>CTATAGAAATGACAGTAAGAACCCTTTAAAAGCCTGCAACTTAGTC<br>TGTAA**GGGG**TCTGTCTCTTAGAGCAGATCCTCA**GG**TATGCTTTAAT<br>AAAGAGTCTTGAGAGTGAACCTT**GG**T**GG**CTACCTGAGTTTTAT**GTG**<br>**GGG**TTTCTGTT**GAGGG**TCC**GGG**CCAGAAACTCTGCA
	FIV Oma	U56928	T**GGG**A**GG**ATT**GG**A**GG**TCCTAAAGACCCTCAGATTGTGATGCTCTTA<br>AACAGAACATTGTAACCTA**GG**AAAATTAAAAACAAAATAGCATGT<br>TAAGAACAGCTGTGTAACCGCAA**GG**CTTAACCACAAACCATATCCG<br>TGCTAAAGTGACGCTTGCTA**GG**CTAGTATGACTCATTTAAGTTTCC<br>AGTAGAATAGTATATAAGAGAAACCTTTAGTCTGTTCA**GGG**CCACT<br>TCTTT**GG**ACTTGCAACTAGCTTGCTA**GGGG**CTTGCTCCTCTGAA**GG**<br>**G**TCCTCA**GG**CACAATAAATTGCTCGTGAGATTTGAACCCTGCCGTG<br>TGTCTGAGTCTTTTCTTTCCTGTGA**GG**CTCC**GG**ATTC**GG**AC**GG**AGA

			CCTTGCA
Ovine-caprine	Visna/Maedi virus	NC_001452.1	**GGG**AAAAGCAGAGTGCTTT**GGAG**AGCTCGAA**GG**AAAGAGTCTCC**G**<br>**GG**CCTCTCCTGCCTGCCTGAAAAGCTCAATAAA**GGAG**TT**GG**CTGAT<br>ATCTGAGCTTGCCTGGTTATTATC**GGG**ATTCGTTACTAATTCCGTG<br>CAACACC**GGAGCGG**ATCTCGCAGC
	CAEV	NC_001463.1	AAAT**GG**AT**GG**CTT**GGAG**AACACCACAAAAATAAAAAAAGAAA**GGG**<br>TGACTGTGAGACAT**GGG**CTAAA**GAGG**ACTAATAACAAGCTA**GG**CC<br>AAATTCCTGTAAATCACTT**GGGGGG**TTATAAGAAAAGCAAGTTCAC<br>TATGACAAAGCAAAATGTAAA**GG**CCAAATTCCTGTAAATCACTT**GG**<br>**GGGG**TTATAAGAAAAGCAAGTTCACTATGACAAAGCAAAATGTAA<br>CCGCAAGTGCTGACAGATGTAACAGCTGACATATCAGCTGATGCTT<br>GCTCATGCTGACACTGTAGCTCTGAGCTGTATATAA**GGAG**AAGCTT<br>GCTGCTTGCACTTCAGAGTTCTA**GGAG**AGTCCCTCCTAGTCTCTCCT<br>CTCC**GAGG**A**GG**TACCGAGACCTCAAAATAAA**GGAG**TGATTGCCTTA<br>CTGCCGAGT**GGAG**AGTGATTACTGA**GCGG**CC**GGTG**TATC**GGGAG**T<br>CGTCCCTTAATCTGTGCAATACCAGA**GCGG**CTCTCGCAGCT**GGCG**C<br>CCAAC**GTGGGG**CCC**GAGG**
	OL	NC_001511.1	TTCCGCTTGTAAACGCTAAATCATGTATCAGCTGATGCTTA**GG**TCA<br>TAACCGCAATTGTAAACAAGTTGCCTATAAAAGCTGCTTGCTAGCT<br>**GGG**AGAGATCAGAGCACTCTT**GGGAGTGG**AAGCTCCCA**GG**TCTCTC<br>CTGCCTGACT**GTGGAG**ACAATAAA**GGAG**TTACTTTACAACTGCGCT<br>AGCCT**GG**TTATTATC**GGG**ATTCGTCACTAATTCTGTGCAACACCAG<br>A**GCGG**ATCTCGCAG
Bovine	BIV	NC_001413.1	T**GTGGGG**CA**GGG**T**GGG**ACCTCA**GG**ACAACAGCAGCCCCC**GG**ACTT<br>CCCATATGTGAATT**GG**ACT**GG**ATCCA**GGG**AACAAAATAACCCAGAA<br>**GGGGG**ATTAGACTCT**GGGG**CTT**GG**TATGAA**GG**CCTGAGA**GG**TTCT<br>CAGTAGATTGTAAGTCTTC**GGCG**AGACTGCATGTCTGCACGTAGAC<br>A**GG**AAATGTTTATCTTCTCAGCTGATT**GTGG**TTA**GG**CCGATTACT**G**<br>**G**AAACTAGACAACCTGATTCATTA**GTGG**TTAAGATTATGCATAAGT<br>GCTCGCAATGATGTAGCTGCTTACGCTTGCTTACTCCGCCCTGAAAC<br>GCCTACCTTAACACGCAACACGCCCACCTGTAAGAATATATAAACC<br>ATATCTTCACTCTGTACTTCAGCTCGTGTAGCTCATTAGCTCCGAGC<br>TCCCCAACCTACAGCCTGAGA**GG**CACT**GG**CTC**GG**TT**GGG**TAGCCAG<br>CCTTTC**GGG**TAATAAA**GG**CTTGTT**GG**CATTC**GG**CATCTACCCGTGC<br>CTCCTGTCTTGTCTTACTCGAGCGAACCCACAACTCCGTCCTGCTGA<br>GCTCACAGCTCGC**GGGGCGG**TGAAGAACACCCAACA

Supp. Table S3. Sequences used for the base conservation analysis of lentiviruses' G4 sequences. Accession numbers of the whole set of sequences were reported for each lentivirus analysed. Accession numbers of reference strains were reported in bold.

Group	Lentivirus	Tot sequences	Accession number
Bovine	JDV	8	**U21603.1**<br>DQ229292.1<br>DQ229290.1<br>DQ229287.1<br>DQ229289.1<br>DQ229291.1<br>DQ229288.1<br>DQ156511.1
Primate	SIVcpz	18	**AF103818.1**<br>AY169968<br>EF535993<br>DQ373065<br>DQ373064<br>DQ373063<br>EF535994<br>DQ373066<br>FR686511<br>AF115393<br>X52154<br>AF382828<br>AF447763<br>EF394357<br>EF394358<br>JQ768416<br>JN091691<br>JN091690
Primate	SIVgor	8	**FJ424864**<br>FJ424863<br>FJ424866<br>FJ424865<br>FJ424871<br>KP004989<br>KP004990<br>KP004991
Primate	SIVmac	15	**M33262**<br>AY033233<br>AY033146<br>AY611495<br>AY611493

			M76764<br>D01065<br>M19499<br>AY600249<br>Y00277<br>BD131285<br>AY576480<br>AY576481<br>M32741<br>MNE027
Primate	SIVsm	60	**JQ864086.1**<br>DQ201174<br>DQ201172<br>DQ201173<br>U72748<br>JQ864085<br>JQ864087<br>JQ864084<br>JX648291<br>JX648292<br>L09211<br>L09213<br>L09212<br>X14307<br>AF077017<br>L03295<br>M31325<br>M80193<br>M80194<br>JX860431<br>AY603050<br>L03298<br>L03297<br>L03296<br>AY221508<br>AY221509<br>AY221513<br>U04991<br>AY221512<br>AY221510<br>AY221511<br>U04989<br>U04990<br>U04986

			U04987<br>U04988<br>U04982<br>U04985<br>U04984<br>U04983<br>M90048<br>AF052649<br>AB553962<br>AB553981<br>AB553921<br>AB553942<br>AB553982<br>AB553973<br>X86725<br>X86728<br>X86729<br>X86726<br>X86730<br>X90853<br>X86731<br>X90854<br>X86732<br>X86724<br>X90851<br>X90850
Primate	SIVver	42	**M29975**<br>M30931<br>X07805<br>DD406200<br>BD092095<br>DD406155<br>DI058170<br>E02137<br>AB253736<br>KR862334<br>KR862349<br>KR862356<br>KR862363<br>KR862345<br>KR862333<br>KR862338<br>KR862335<br>KR862354

			KR862362<br>KR862347<br>KR862348<br>KR862343<br>KR862355<br>KR862360<br>KR862339<br>KR862346<br>KR862350<br>KR862341<br>KR862344<br>KR862357<br>KR862353<br>KR862336<br>KR862361<br>KR862331<br>KR862358<br>KR862332<br>KR862359<br>KR862340<br>KR862337<br>KR862352<br>KR862351<br>KR862342
Primate	HIV-2	24	**NC_001722.1**<br>U38293<br>M30502<br>U22047<br>M30895<br>J04542<br>J04498<br>AY509259<br>AY509260<br>J03654<br>D00835<br>EU980602<br>DQ307022<br>M15390<br>L07625<br>AB485670<br>U27200<br>X61240<br>AB100245<br>AF208027

			EU02834<br>L36874<br>AB499693<br>AB499694
Primate	HIV-1 M	1106	**NC_001802.1**<br>AB023804<br>AB049811<br>AB052600<br>AB052601<br>AB052602<br>AB052603<br>AB052604<br>AB052605<br>AB052606<br>AB052607<br>AB052608<br>AB052609<br>AB052610<br>AB052611<br>AB052612<br>AB052867<br>AB052995<br>AB070352<br>AB097868<br>AB097869<br>AB097872<br>AB098330<br>AB098331<br>AB098332<br>AB098333<br>AB195665<br>AB195666<br>AB195667<br>AB220944<br>AB220945<br>AB220946<br>AB220947<br>AB220948<br>AB221005<br>AB221125<br>AB221126<br>AB231893<br>AB231894<br>AB231895

				AB231896<br>AB231897<br>AB231898<br>AB253421<br>AB253422<br>AB253423<br>AB253424<br>AB253425<br>AB253426<br>AB253427<br>AB253428<br>AB253429<br>AB253430<br>AB253431<br>AB253432<br>AB253635<br>AB253636<br>AB253637<br>AB253638<br>AB253639<br>AB253640<br>AB253641<br>AB253642<br>AB253643<br>AB253644<br>AB253645<br>AB253646<br>AB253647<br>AB253648<br>AB253649<br>AB253650<br>AB253651<br>AB253652<br>AB253653<br>AB253654<br>AB253655<br>AB253656<br>AB253657<br>AB253658<br>AB253659<br>AB253660<br>AB253661<br>AB253662<br>AB253663

			AB253664
			AB253665
			AB253666
			AB253667
			AB253668
			AB253669
			AB253670
			AB253671
			AB253672
			AB253673
			AB253674
			AB253675
			AB253676
			AB253677
			AB253678
			AB253679
			AB253680
			AB253681
			AB253682
			AB253683
			AB253684
			AB253685
			AB253686
			AB253687
			AB253688
			AB253689
			AB253690
			AB253691
			AB253692
			AB253695
			AB253696
			AB253697
			AB253698
			AB253699
			AB253700
			AB253701
			AB253702
			AB253703
			AB253704
			AB253705
			AB253706
			AB253707
			AB253708
			AB253709

				AB253710<br>AB253711<br>AB253712<br>AB253713<br>AB253714<br>AB253715<br>AB253716<br>AB253717<br>AB253718<br>AB253719<br>AB253720<br>AB253721<br>AB253722<br>AB253723<br>AB253725<br>AB254141<br>AB254142<br>AB254143<br>AB254144<br>AB254145<br>AB254146<br>AB254147<br>AB254148<br>AB254149<br>AB254150<br>AB254151<br>AB254152<br>AB254153<br>AB254154<br>AB254155<br>AB254156<br>AB286849<br>AB286850<br>AB286851<br>AB286852<br>AB286853<br>AB286854<br>AB286855<br>AB286856<br>AB286857<br>AB286858<br>AB286859<br>AB286860<br>AB286862

				AB286863<br>AB286864<br>AB286955<br>AB286956<br>AB287003<br>AB287004<br>AB287363<br>AB287364<br>AB287365<br>AB287366<br>AB287367<br>AB287368<br>AB287369<br>AB287370<br>AB287371<br>AB287372<br>AB287376<br>AB287377<br>AB287378<br>AB287379<br>AB289587<br>AB289588<br>AB289589<br>AB289590<br>AB480047<br>AB480048<br>AB480298<br>AB480299<br>AB480300<br>AB480301<br>AB480692<br>AB480693<br>AB480698<br>AB485632<br>AB485633<br>AB485634<br>AB485635<br>AB485636<br>AB485637<br>AB485643<br>AB485644<br>AB485645<br>AB485646<br>AB485647

				AB485648<br>AB485649<br>AB485650<br>AB485651<br>AB485652<br>AB485653<br>AB485654<br>AB485655<br>AB485656<br>AB485657<br>AB485658<br>AB485659<br>AB485662<br>AB485663<br>AB564744<br>AB564745<br>AB564746<br>AB565478<br>AB565479<br>AB565495<br>AB565496<br>AB565497<br>AB565498<br>AB565499<br>AB565500<br>AB565501<br>AB565502<br>AB565503<br>AB565504<br>AB604946<br>AB604947<br>AB604948<br>AB604949<br>AB604951<br>AB641836<br>AB641837<br>AB646289<br>AB646691<br>AB731663<br>AB731664<br>AB731665<br>AB731666<br>AB731667<br>AB731668

				AB731669<br>AB731670<br>AB746344<br>AB746345<br>AB773884<br>AB773885<br>AF003887<br>AF004394<br>AF023427<br>AF023428<br>AF023429<br>AF023430<br>AF023431<br>AF023432<br>AF023433<br>AF023434<br>AF023435<br>AF023436<br>AF023437<br>AF023438<br>AF023439<br>AF023440<br>AF023441<br>AF025394<br>AF042100<br>AF042101<br>AF063142<br>AF063143<br>AF063144<br>AF063145<br>AF063146<br>AF063147<br>AF063149<br>AF063150<br>AF063151<br>AF063152<br>AF063153<br>AF063154<br>AF063155<br>AF063156<br>AF063157<br>AF063158<br>AF063159<br>AF063160

				AF063161<br>AF063162<br>AF063163<br>AF063164<br>AF063165<br>AF063166<br>AF063167<br>AF063168<br>AF063169<br>AF063170<br>AF063174<br>AF063175<br>AF063176<br>AF063177<br>AF063178<br>AF063179<br>AF063180<br>AF063181<br>AF063182<br>AF063183<br>AF063184<br>AF063185<br>AF064699<br>AF069140<br>AF070521<br>AF080159<br>AF080160<br>AF080161<br>AF080162<br>AF080163<br>AF080164<br>AF080165<br>AF080166<br>AF080167<br>AF080168<br>AF086817<br>AF096778<br>AF096779<br>AF096780<br>AF096781<br>AF096782<br>AF119819<br>AF119820<br>AF127566

				AF127567<br>AF127568<br>AF127569<br>AF127570<br>AF127571<br>AF127572<br>AF127573<br>AF164485<br>AF169762<br>AF169763<br>AF169764<br>AF169765<br>AF169766<br>AF169767<br>AF169768<br>AF169769<br>AF169770<br>AF169771<br>AF169772<br>AF169773<br>AF169774<br>AF169776<br>AF169777<br>AF169779<br>AF169780<br>AF169781<br>AF169782<br>AF169783<br>AF169784<br>AF169785<br>AF169786<br>AF169787<br>AF169788<br>AF169789<br>AF196708<br>AF196709<br>AF196710<br>AF196711<br>AF196712<br>AF196714<br>AF196715<br>AF196716<br>AF196717<br>AF196718

				AF196719<br>AF196721<br>AF196722<br>AF196723<br>AF196724<br>AF196725<br>AF196726<br>AF196727<br>AF196730<br>AF196731<br>AF196732<br>AF196733<br>AF196734<br>AF196736<br>AF196737<br>AF196738<br>AF196740<br>AF196741<br>AF196742<br>AF196743<br>AF196744<br>AF196745<br>AF196746<br>AF196747<br>AF196748<br>AF196750<br>AF196752<br>AF196753<br>AF196754<br>AF196755<br>AF196756<br>AF196757<br>AF196759<br>AF196760<br>AF196761<br>AF196762<br>AF196763<br>AF196764<br>AF196765<br>AF196766<br>AF196767<br>AF256204<br>AF256205<br>AF256206

				AF256207
				AF256208
				AF256209
				AF256210
				AF256211
				AF259954
				AF259955
				AF272004
				AF272006
				AF286365
				AF290027
				AF290028
				AF290029
				AF290030
				AF290031
				AF321523
				AF385934
				AF385935
				AF385936
				AF411964
				AF411965
				AF411966
				AF411967
				AF413987
				AF414006
				AF538302
				AF538303
				AF538304
				AF538305
				AF538306
				AF538307
				AJ237565
				AJ245481
				AJ288981
				AJ288982
				AJ291718
				AJ291719
				AJ291720
				AJ508595
				AJ508596
				AJ508597
				AJ866553
				AJ866554
				AJ866556

			AJ866557
			AJ866558
			AM000053
			AM000055
			AM851091
			AX032749
			AX316202
			AX455917
			AX455929
			AX772015
			AY162223
			AY162224
			AY162225
			AY228556
			AY228557
			AY352275
			AY445524
			AY452651
			AY452652
			AY452653
			AY452654
			AY452655
			AY452656
			AY452657
			AY452658
			AY452659
			AY452660
			AY452661
			AY835748
			AY835749
			AY835750
			AY835751
			AY835752
			AY835753
			AY835754
			AY835755
			AY835756
			AY835757
			AY835758
			AY835759
			AY835760
			AY835761
			AY835762
			AY835763

				AY835764<br>AY835765<br>AY835766<br>AY835767<br>AY835768<br>AY835769<br>AY835770<br>AY835771<br>AY835772<br>AY835773<br>AY835774<br>AY835775<br>AY835776<br>AY835777<br>AY835778<br>AY835779<br>AY835780<br>AY835781<br>AY860947<br>AY970950<br>BD187399<br>BD238372<br>BD410064<br>BD437615<br>BD437626<br>CS272319<br>D10112<br>D86068<br>D86069<br>DD033495<br>DD207211<br>DQ007901<br>DQ007902<br>DQ007903<br>DQ083238<br>DQ837381<br>DQ848354<br>DQ848355<br>DQ848356<br>DQ848357<br>DQ848358<br>DQ848359<br>DQ848360<br>DQ848361

				DQ848362<br>DQ848363<br>DQ848365<br>DQ848366<br>DQ848368<br>DQ848369<br>DQ848370<br>DQ848371<br>DQ848373<br>DQ848374<br>DQ848376<br>DQ848377<br>DQ848379<br>DQ848380<br>DQ848381<br>DQ848382<br>DQ848383<br>DQ848385<br>DQ848386<br>DQ848388<br>DQ848389<br>DQ848390<br>DQ848391<br>DQ848392<br>DQ848393<br>DQ848394<br>DQ848395<br>DQ848396<br>DQ848397<br>DQ848398<br>DQ848399<br>DQ848400<br>DQ848401<br>DQ848402<br>DQ848403<br>DQ848404<br>DQ848405<br>DQ848406<br>DQ848407<br>DQ848408<br>DQ848409<br>DQ848410<br>DQ848411<br>DQ848412

			DQ848413	
			DQ848414	
			DQ848415	
			DQ848416	
			DQ848417	
			DQ848418	
			DQ848420	
			DQ848421	
			DQ848422	
			DQ848423	
			DQ848424	
			DQ848425	
			DQ848426	
			DQ848427	
			DQ848428	
			DQ848429	
			DQ848430	
			DQ848431	
			DQ848432	
			DQ848433	
			DQ848434	
			DQ848435	
			DQ848436	
			DQ848437	
			DQ848438	
			DQ848439	
			DQ848440	
			DQ848441	
			DQ848442	
			DQ848443	
			DQ848444	
			DQ848445	
			DQ848446	
			DQ848447	
			DQ848448	
			DQ848449	
			DQ848450	
			DQ848451	
			DQ848452	
			DQ848453	
			DQ848454	
			DQ848455	
			DQ848456	
			DQ848457	

				DQ848458<br>DQ848459<br>DQ848460<br>DQ848461<br>DQ848462<br>DQ848463<br>DQ848464<br>DQ848465<br>DQ848466<br>DQ848467<br>DQ848468<br>DQ848469<br>DQ848470<br>DQ848471<br>DQ848472<br>DQ848473<br>DQ848474<br>DQ848475<br>DQ848476<br>DQ848477<br>DQ848478<br>DQ848479<br>DQ848480<br>DQ848481<br>DQ848482<br>DQ848483<br>DQ848484<br>DQ848485<br>DQ848486<br>DQ848487<br>DQ848488<br>DQ848489<br>DQ848490<br>DQ848491<br>DQ848492<br>DQ848493<br>DQ848494<br>DQ848495<br>DQ848496<br>DQ848497<br>DQ848498<br>DQ848499<br>DQ848500<br>DQ848501

				DQ848502<br>DQ848503<br>DQ848504<br>DQ848505<br>DQ848506<br>DQ848507<br>DQ848508<br>DQ848509<br>DQ848510<br>DQ848511<br>DQ848512<br>DQ848513<br>DQ848514<br>DQ848515<br>DQ848516<br>DQ848517<br>DQ848518<br>DQ848519<br>DQ848520<br>DQ848521<br>DQ848522<br>DQ848523<br>DQ848524<br>DQ848525<br>DQ848526<br>DQ848527<br>DQ848528<br>DQ848529<br>DQ848530<br>DQ848531<br>DQ848532<br>DQ848533<br>DQ848534<br>DQ848535<br>DQ848536<br>DQ848537<br>DQ848538<br>DQ848539<br>DQ848540<br>DQ848541<br>DQ848542<br>DQ848543<br>DQ848544<br>DQ848545

				DQ848546<br>DQ848547<br>DQ848548<br>DQ848549<br>DQ848550<br>DQ848551<br>DQ848552<br>DQ848553<br>DQ848554<br>DQ848555<br>DQ848556<br>DQ848557<br>DQ848558<br>DQ848559<br>DQ848560<br>DQ848561<br>DQ848562<br>DQ848563<br>DQ854714<br>DQ854715<br>DQ854716<br>DQ859178<br>DQ859179<br>DQ859180<br>DQ990880<br>E12793<br>EF036527<br>EF036528<br>EF036529<br>EF036530<br>EF036531<br>EF036532<br>EF036533<br>EF036534<br>EF036535<br>EF036536<br>EF057102<br>EF178613<br>EF363122<br>EF363123<br>EF363124<br>EF363125<br>EF363126<br>EF363127

				EF469243<br>EF592600<br>EF592601<br>EF592602<br>EF592603<br>EF592604<br>EF592605<br>EU293444<br>EU293445<br>EU293446<br>EU293447<br>EU293448<br>EU293449<br>EU293450<br>EU448295<br>EU448296<br>EU861977<br>FB341548<br>FB675252<br>FB707281<br>FJ210864<br>FJ210865<br>FJ210866<br>FJ210867<br>FJ210868<br>FJ210869<br>FM877777<br>FM877778<br>FM877779<br>FM877781<br>FM877782<br>FM877783<br>FW555696<br>GQ386774<br>GQ386775<br>GQ386776<br>GQ386778<br>GQ386779<br>GQ386780<br>GQ386781<br>GQ386782<br>GQ386783<br>GQ386784<br>GQ386785

				GQ386786
				GQ386787
				GQ386788
				GQ386789
				GQ386790
				GQ386791
				GQ386792
				GQ386793
				GQ386794
				GQ386795
				GU177863
				GU647196
				GU647197
				GU647198
				GU733713
				GU733714
				GU733715
				GU733716
				GU733717
				HH978650
				HM100716
				HM215249
				HM215251
				HM469972
				HM469973
				HM469974
				HM469975
				HM469976
				HM469977
				HM469978
				HM469979
				HM469980
				HM469981
				HM469982
				HM469983
				HV199808
				JA806687
				JF719819
				JF939054
				JN188292
				JN944897
				JN944905
				JN944907
				JN944909

				JN944911<br>JN944917<br>JN944928<br>JN944930<br>JN944936<br>JN944938<br>JN944939<br>JN944940<br>JN944941<br>JN944943<br>JN944944<br>JN944945<br>JN944946<br>JN944947<br>JN944948<br>JQ268646<br>JQ268647<br>JQ268656<br>JQ268657<br>JQ268658<br>JQ268660<br>JQ268688<br>JQ268689<br>JQ268690<br>JQ268691<br>JQ268725<br>JQ268731<br>JX112869<br>JX236668<br>JX236669<br>JX236670<br>JX236671<br>JX236672<br>JX236673<br>JX236674<br>JX236675<br>JX236676<br>JX236677<br>JX236678<br>JX236679<br>JX289943<br>JX289944<br>JX289945<br>JX289946

				JX289947<br>JX289949<br>JX289950<br>JX289951<br>JX289952<br>JX289953<br>JX289954<br>JX289955<br>JX289956<br>JX289957<br>JX289958<br>JX289960<br>JX289961<br>JX289962<br>JX289964<br>JX289965<br>JX289966<br>JX289967<br>JX289968<br>JX289969<br>JX289970<br>JX289971<br>JX289972<br>JX289973<br>JX289974<br>JX289975<br>JX289976<br>JX289977<br>JX289978<br>JX289979<br>JX289980<br>JX289981<br>JX289982<br>JX289983<br>JX289984<br>JX289985<br>JX289986<br>JX289988<br>JX289989<br>JX289991<br>JX289992<br>JX289993<br>JX289994<br>JX289995

				JX289996
				JX289997
				JX289998
				JX289999
				JX290000
				JX290001
				JX290002
				JX290004
				JX290006
				JX290007
				JX290008
				JX290010
				JX290011
				JX290012
				JX290013
				JX290014
				JX290015
				JX290016
				JX290017
				JX290018
				JX290019
				JX290020
				JX290021
				JX290022
				JX290023
				JX290024
				K02007
				K02083
				K03455
				KC156114
				KC156115
				KC156116
				KC156117
				KC156118
				KC156119
				KC156120
				KC156121
				KC156122
				KC156123
				KC156124
				KC156125
				KC156126
				KC156127
				KC156128

				KC156129
				KC156130
				KC156210
				KC156211
				KC156212
				KC156213
				KC156214
				KC156215
				KC156216
				KC156217
				KC156218
				KC156219
				KC156220
				KC156221
				KC197029
				KC197030
				KC197031
				KC197032
				KC197033
				KC492737
				KC492738
				KC503854
				KC503855
				KC899079
				KC899080
				KC899081
				KC911635
				KC911637
				KC911639
				KC911640
				KC911641
				KC911642
				KC911644
				KC914396
				KF234628.
				KF766537
				KF766538
				KF766539
				KF766540
				KF766541
				KF766542
				KF990605
				KF990606
				KF990607

				KF990608
				KJ925006
				KP170487
				KT152840
				KT152842
				KU168256
				KU168257
				KU168258
				KU168259
				KU168261
				KU168266
				KU168269
				KU168271
				KU168272
				KU168273
				KU168274
				KU168275
				KU168277
				KU168299
				KU168301
				KU168302
				KU168304
				KU168305
				KU168306
				KU168308
				KU168309
				KU168310
				KU168311
				KU641402
				L32865
				M13136
				M15653
				M17449
				M22639
				M38429
				M38431
				M66534
				M93258
				M93259
				S63986
				S76651
				U12055
				U21135
				U34603

			U34604<br>U39362<br>U51188<br>U51189<br>U63535<br>U63536<br>U63537<br>U63538<br>U63539<br>X01762
Primate	HIV-1 N	7	**DQ017383**<br>AJ006022<br>AJ271370<br>AY532635<br>FB675251<br>HV199807<br>JN572926
Primate	HIV-1 O	46	**L20587.1**<br>AB485666<br>AB485667<br>AB485668<br>AB485669<br>AF407418<br>AJ302646<br>AJ302647<br>AY169802<br>AY169803<br>AY169804<br>AY169805<br>AY169807<br>AY169808<br>AY169809<br>AY169810<br>AY169811<br>AY169812<br>AY169813<br>AY169815<br>AY169816<br>AY618998<br>AY623602<br>JX245014<br>JX245015<br>KF859742

			KF859743<br>KF859744<br>KU168281<br>KU168282<br>KU168283<br>KU168284<br>KU168285<br>KU168286<br>KU168288<br>KU168289<br>KU168290<br>KU168291<br>KU168292<br>KU168293<br>KU168294<br>KU168295<br>KU168296<br>KU168297<br>KU168298<br>L20571
